# Impact of Bacterial Genetics on the Transmission of Isoniazid-Resistant Mycobacterium tuberculosis

**DOI:** 10.1371/journal.ppat.0020061

**Published:** 2006-06-16

**Authors:** Sebastien Gagneux, Marcos V Burgos, Kathryn DeRiemer, Antonio Enciso, Samira Muñoz, Phillip C Hopewell, Peter M Small, Alexander S Pym

**Affiliations:** 1 Division of Infectious Diseases and Geographic Medicine, Stanford University Medical Center, Stanford, California, United States of America; 2 Institute for Systems Biology, Seattle, Washington, United States of America; 3 Department of Internal Medicine, Division of Infectious Diseases, University of New Mexico, Albuquerque, New Mexico, United States of America; 4 Veterans Affairs Medical Center, Albuquerque, New Mexico, United States of America; 5 School of Medicine, University of California Davis, Davis, California, United States of America; 6 Unidad de Investigacion Medica de Zacatecas, Zacatecas, Mexico; 7 Division of Pulmonary and Critical Care Medicine, University of California San Francisco, San Francisco, California, United States of America; 8 Bill and Melinda Gates Foundation, Seattle, Washington, United States of America; 9 Unit for Clinical and Biomedical TB Research, South African Medical Research Council, Durban, South Africa; University of Washington, United States of America

## Abstract

Understanding the ecology of drug-resistant pathogens is essential for devising rational programs to preserve the effective lifespan of antimicrobial agents and to abrogate epidemics of drug-resistant organisms. Mathematical models predict that strain fitness is an important determinant of multidrug-resistant Mycobacterium tuberculosis transmission, but the effects of strain diversity have been largely overlooked. Here we compared the impact of resistance mutations on the transmission of isoniazid-resistant M. tuberculosis in San Francisco during a 9-y period. Strains with a KatG S315T or *inhA* promoter mutation were more likely to spread than strains with other mutations. The impact of these mutations on the transmission of isoniazid-resistant strains was comparable to the effect of other clinical determinants of transmission. Associations were apparent between specific drug resistance mutations and the main M. tuberculosis lineages. Our results show that in addition to host and environmental factors, strain genetic diversity can influence the transmission dynamics of drug-resistant bacteria.

## Synopsis

Resistance to antibiotics is an increasing global public health problem. Multidrug-resistant tuberculosis is threatening the control of this deadly infectious disease in many parts of the world. Studies to identify the factors that lead to the emergence and subsequent spread of drug-resistant organisms have clearly shown that patient adherence to a 6 mo course of treatment is very important to prevent the development of drug-resistant tuberculosis. However, little is known about how bacterial genetics might influence the spread of drug-resistant strains. This study showed that among the strains in San Francisco with resistance to isoniazid, one of the main anti-tuberculosis drugs, only certain strains were transmitted effectively. The transmission of specific isoniazid-resistant strains depended on the type of genetic mutation leading to resistance. Also, isoniazid-resistant strains coming from different parts of the world were associated with different resistance mutations. This study's results show that the spread of drug resistance is due to the strain of tuberculosis as well as the environment and characteristics of the patient, and may partially explain why some areas of the world have worse drug resistance problems than others.

## Introduction

Drug resistance in Mycobacterium tuberculosis has been encountered worldwide [1]. It has been postulated and mathematical models suggest that drug resistance will seriously impair global tuberculosis control efforts [2,3]. In at least some areas, resistant strains are replacing susceptible ones, leading to “hotspots” of drug resistance [4]. In other areas the introduction of standard control programs has interrupted transmission of drug-resistant strains [5]. Thus, it is important to increase our understanding of the biology of drug resistance to predict and prevent the future spread of resistant strains.

The factors that determine the epidemiology of drug-resistant organisms that can be influenced by human behavior, such as patterns of antimicrobial usage, have been studied extensively. There has been less analysis of the role of intrinsic microbiologic factors, such as the relative fitness of drug-resistant strains. Ecological theory and mathematical models suggest that the relative fitness of drug-resistant strains can profoundly affect the emergence and propagation of these organisms [2,3,6,7].

In vitro models using non-pathogenic organisms have shown that drug resistance–conferring mutations are usually associated with a fitness cost [8]. The degree to which the mutation affects the fitness of the organism varies with the specific drug resistance mutation, the environment, and genetic background of the strain [8–12]. Compensatory evolution can ameliorate the fitness effects of drug resistance mutations [13–16]. However, it is unclear if such experimental models can be extrapolated to explain the behavior of pathogenic drug-resistant bacteria in human populations.

To date, epidemiological studies and surveillance of M. tuberculosis have not fully resolved whether the transmission dynamics of drug-resistant strains are different from drug-sensitive strains. Most molecular epidemiological work has focused on multidrug-resistant strains (resistant to at least isoniazid and rifampin) of M. tuberculosis. In such studies, multidrug-resistant strains have been associated with both more and less ongoing transmission than sensitive strains [17–20]. These discrepancies suggest that drug-resistant strains may be heterogeneous and that drug resistance–conferring mutations might have a variable impact on strain fitness. In the case of isoniazid resistance, there is some laboratory and epidemiological evidence in support of this [21–24]. Alternatively, the genetic background of a strain might influence how it adapts to antibiotics.

In this study, we have used comprehensive molecular epidemiologic data from San Francisco to examine the role of specific resistance-conferring mutations and strain genetic background on the transmission dynamics of isoniazid-resistant M. tuberculosis in a human population.

## Results

### Bacterial Strains and Resistance Profiles

This study was based on an ongoing population-based molecular epidemiological study of tuberculosis transmission in San Francisco [25]. For the study period, 1991–1999, 2,104 cases of tuberculosis were culture positive for M. tuberculosis. Full drug susceptibility testing was performed on 2,081 (98.9%) isolates, which were all recovered from distinct tuberculosis patients. Of these isolates, 298 (14.3%) were initially resistant to at least one first-line anti-tuberculosis drug (Table 1). Resistance to isoniazid was the most common form of drug resistance. Genetic characterization was available for 152 (85.9%) of the 177 isoniazid-resistant isolates. Among these 152 isolates with resistance to isoniazid, with or without resistance to other drugs, 136 had unique restriction fragment length polymorphism (RFLP) patterns. 16 isolates (10.5%) shared an RFLP pattern, drug resistance phenotype, drug resistance–conferring mutation, and lineage specific marker (see below) with at least one other isolate, indicating they were linked in seven genetically distinct isolate clusters. Therefore, there were a total of 143 genetically distinct isoniazid-resistant strains (clones), providing a large, genetically well- characterized and representative group of strains from a defined human population for studying the biology of resistance.

**Table 1:**
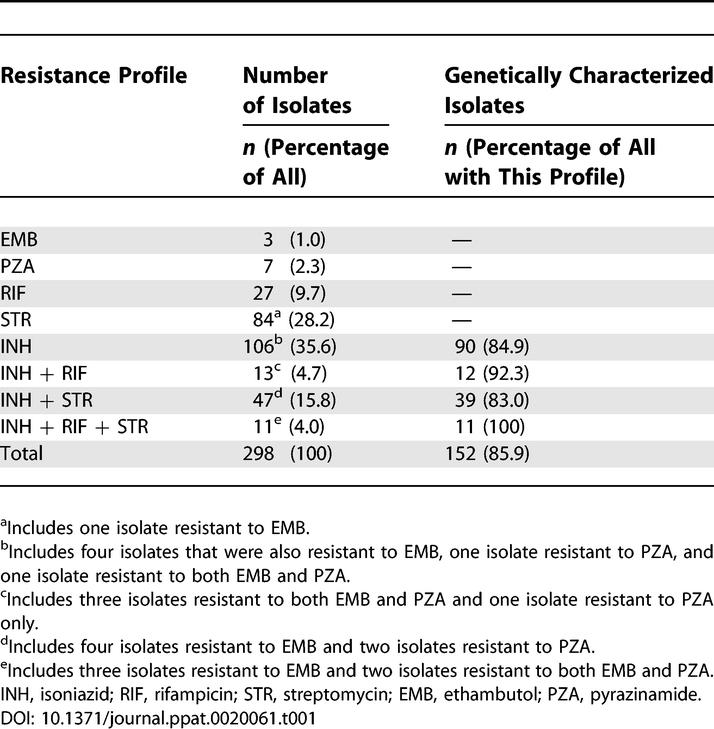
Primary Drug-Resistant Clinical Isolates of M. tuberculosis Isolated in San Francisco from 1991–1999

### Drug-Resistant Alleles

Resistance to isoniazed has been associated with various chromosomal mutations, but those in *katG,* and in the promoter regions of *inhA,* occur most frequently in clinical isolates of M. tuberculosis [26,27]. *katG* encodes a catalase-peroxidase that activates the prodrug isoniazid to its active form and also acts as a virulence factor, protecting against oxidative stress [28]. InhA, encoded by *inhA,* is a component of FAS-II (fatty acid elongation system) that is required for mycolic acid synthesis and is a target for isoniazid [29–31]. *inhA* promoter mutations result in isoniazid resistance through up-regulation of *inhA*. Mutations in the *ahpC* promoter have also been described in isoniazid-resistant isolates, but are thought to be compensatory, helping strains adjust to the additional oxidative stress imposed by mutations in *katG* [32–34].

Sequencing of these three principal isoniazid resistance loci of the 143 isoniazid-resistant strains revealed that 128 (89.5%) had at least one mutation in either the *katG* gene or in the *inhA* promoter region (Table 2). Only 16 (11.2%) of these strains had no resistance mutations in the regions sequenced. After excluding the KatG L463R substitution, a polymorphism not associated with isoniazid resistance [35], 92 strains (64.3%) were found to have *katG* gene mutations. 58 (40.6%) strains had the S315T mutation and a further 34 (23.8%) had other mutations affecting *katG,* including three strains with complete gene deletions. *inhA* promoter mutations were detected in 46 (32.2%) strains, of which 11 (7.7%) also had a *katG* mutation. Characterization of the *ahpC* promoter regions showed that mutations in this region were uncommon, occurring in only 11 (7.7%) strains (Table 2). Pertinently, none of these arose in strains without other isoniazid resistance–conferring mutations, and ten of the 11 strains with mutations in the *ahpC* promoter region had mutations in *katG,* including two of the strains with complete gene deletions. These findings support the notion that *ahpC* promoter mutations are in fact compensatory, rather than causing resistance directly.

**Table 2:**
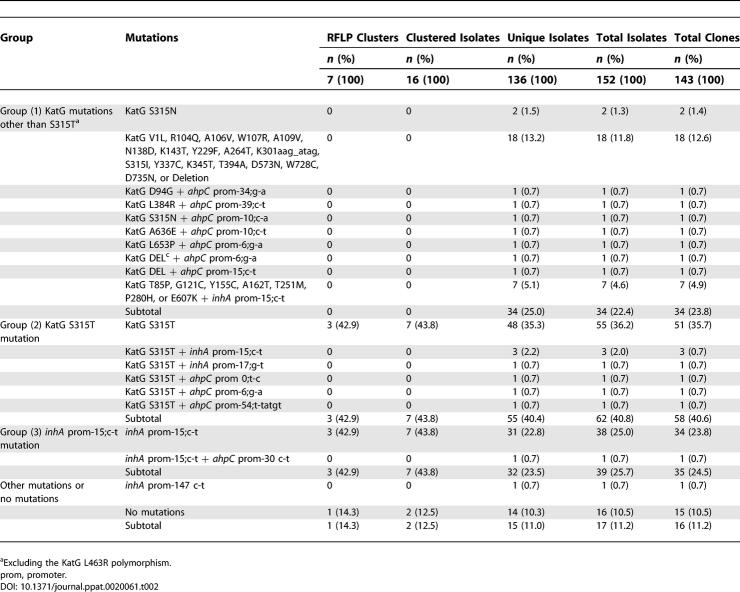
Isoniazid Resistance–Conferring Mutations and RFLP Clusters Identified in 152 Isoniazid-Resistant Isolates of M. tuberculosis from San Francisco, 1991–1999

### Transmission Dynamics of Drug Resistance–Conferring Mutations

Genetic clustering has been used extensively in molecular epidemiological studies as a measure for ongoing tuberculosis transmission [36,37]. The underlying assumption is that in a strict population-based study such as the one reported here, tuberculosis patients harboring isolates with unique RFLP patterns represent cases of reactivation of a latent infection, whereas patients with genetically clustered isolates are epidemiologically linked and represent chains of tuberculosis transmission. This is based on more than a decade of molecular epidemiological studies carried out in many communities from different countries. Outbreaks of tuberculosis in health-care facilities, prisons, and other contained situations have been shown to be caused by genetically identical strains [38,39]. In addition, epidemiological links have been demonstrated between genetically clustered strains identified at the community level [40]. For example, a study from Holland could establish an epidemiological link for 86% of genetically clustered strains [41].

To determine the relationship between drug resistance mutations and tuberculosis transmission dynamics, we compared the percentage of genetic clustering in isolates associated with the different drug resistance mutations (Table 2). Genetic clustering is usually based only on specific RFLP fingerprinting patterns. However, by including the phenotypic drug resistance profile, the specific drug resistance mutation, as well as the markers for the major M. tuberculosis lineage (see below), we ensured a more stringent definition of genetic clustering. The isoniazid-resistant isolates with resistance mutations could be divided into three groups (Table 2): those isolates with a mutation in KatG other than the S315T (Group 1), those isolates with a KatG S315T mutation (Group 2), and those isolates with a *inhA*-15;c-t promoter mutation only (Group 3). Whereas isolates with KatG mutations other than S315T were never genetically clustered (Table 2), 11.3% of the KatG S315T mutants (Fisher's 2-tailed exact test: *p* < 0.05) and 17.8% of the *inhA*-15;c-t promoter mutants (Fisher's 2-tailed exact test: *p* < 0.002) showed genetic clustering (Table 2). 11.7% of strains without a defined resistance mutation were genetically clustered. No strains with *ahpC* promoter mutations were genetically clustered.

These data demonstrate that only two isoniazid resistance mutations were effectively propagated in San Francisco during the study period. This is consistent with the role of catalase-peroxidase (KatG) in the pathogenicity of *M. tuberculosis,* which is a known virulence factor [23,42]. KatG S315T is an unusual substitution resulting in a functional enzyme that retains catalase-peroxidase activity but with a diminished capacity to activate isoniazid [23,43,44]. Similarly, the *inhA*-15;c-t promoter mutation does not affect KatG activity. This is in contrast to the other substitutions in, or deletions of, *katG,* which have a more profound effect on enzyme activity [23,42], although all of the mutations identified in this study have not been empirically evaluated. Thus, KatG activity could explain the different transmission dynamics of the various mutations.

Because resistance to additional drugs could influence the fitness of isoniazid-resistant strains in an unpredictable manner, we repeated our analysis on the 90 isolates resistant to isoniazid alone (Table 1). We found that 7/28 (25.0%) of the isolates with the KatG S315T mutation and 4/23 (17.4%) with the *inhA*-15;c-t were genetically clustered. The comparisons to the group of isolates with other *katG* mutations remained statistically significant (Fisher's 2-tailed exact test: *p* < 0.02 and *p* < 0.05, respectively).

To assess the importance of specific mutations for the propagation of isoniazid-resistant strains, compared to other factors generally considered in conventional molecular epidemiologic studies, we performed univariate and multivariate analyses. The univariate analysis showed that isoniazid resistance due to mutations from Group 2 or 3, which are likely to maintain KatG activity, was the third strongest risk factor for genetic clustering (Table 3). In the final multivariate logistic regression model, these mutations perfectly predicted genetic clustering because all genetically clustered isolates had Group 2 or 3 mutations. Only homelessness remained an independent risk factor for genetic clustering (adjusted odds ratio = 9.6, 95% confidence interval: 1.7–53.3, *p* = 0.01). Taken together, these results indicate that specific drug resistance mutations have an impact on the successful propagation of isoniazid-resistant tuberculosis, as great or greater than other well-recognized risk factors, such as the presence of acid-fast bacilli in sputum smears.

**Table 3:**
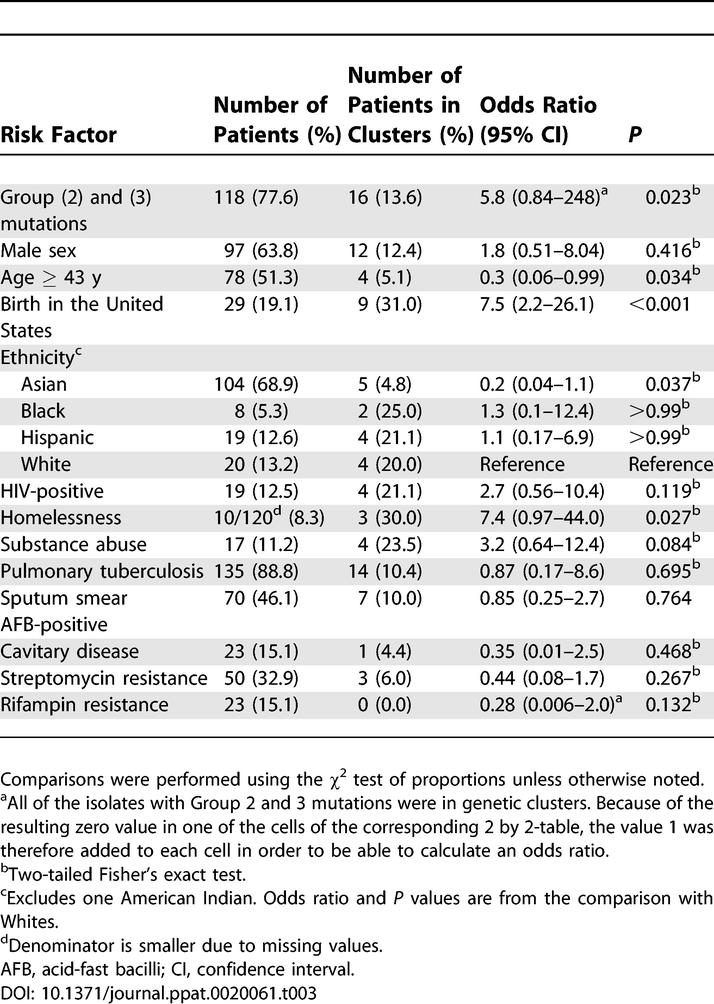
Results of the Univariate Analysis Showing Host and Bacterial Factors Associated with Clustering in 152 Isoniazid-Resistant Isolates of M. tuberculosis from San Francisco

### Strain Lineage and Drug Resistance–Conferring Mutations

The global population structure of M. tuberculosis consists of six main phylogenetic lineages [45]. These lineages can be defined by single nucleotide polymorphisms and large sequence polymorphisms, which in M. tuberculosis behave as unique event polymorphisms, and therefore allow for robust phylogenetic inference [46,47]. To evaluate the influence of strain genetic background on the genetics of resistance, associations between strain lineage and isoniazid resistance–conferring mutations were sought. All of the 152 isoniazid-resistant isolates were screened for lineage-specific genetic markers and classified as belonging to the East-Asian lineage, the Indo-Oceanic lineage, or the Euro-American lineage [45]. Of the strains examined in this way, 37 (24.3%) belonged to the East-Asian lineage, 51 (33.6%) to the Indo-Oceanic lineage, and 64 (42.1%) to the Euro-American lineage. After exclusion of ten isolates harboring a combination of *katG* and *inhA* promoter mutations (Table 2), we found statistically significant associations between the three lineages and the three groups of isoniazid-resistance mutations (Table 4). The East-Asian lineage was associated with KatG mutations other than S315T, the *inhA* promoter mutation occurred significantly more frequently among the Indo-Oceanic lineage, and the Euro-American lineage was associated with the KatG S315T mutation. These associations suggest that genetic differences between the strain lineages could influence their propensity for different mutations.

**Table 4:**
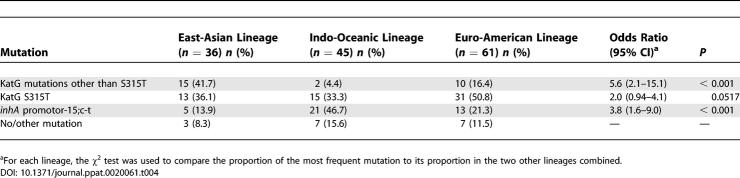
Association between Three M. tuberculosis Strain Lineages and Three Classes of Isoniazid Resistance–Conferring Mutations

We previously showed that within San Francisco, strains belonging to the Euro-American lineage were more likely to result in secondary cases than strains from other lineages [45]. In order to control for this potential confounding factor, we repeated our analysis of transmission and included only the 64 isolates belonging to the Euro-American lineage. We found that 14 out of 52 isolates (26.9%) with isoniazid-resistance mutations from Group 2 and 3 were genetically clustered, whereas none of the 12 isolates with other *katG* mutations were genetically clustered (Fisher's 2-tailed exact test*: p* = 0.0539).

## Discussion

Isoniazid resistance is caused by several distinct mechanisms, and data from animal models indicate there are variations in the pathogenicity of isoniazid-resistant strains [22,23]. As reported here, differences are also apparent in human tuberculosis, as among mutations in KatG only the S315T mutation was associated with secondary cases. In contrast, strains with other mutations in *katG* did not produce a single secondary case in the population during the 9-y study period. Catalase-peroxidase encoded by *katG* transforms the prodrug isoniazid into its bioactive form, as well as protecting bacteria against oxidative stress encountered during host infection [28]. Loss of KatG activity is therefore usually associated with reduced virulence [23]. Our data are consistent with the findings of in vitro and in vivo experiments showing that isogenic mutants with the KatG S315T substitution retain KatG activity and associated virulence in mice [23,43,44]. Recently, the crystal structure of KatG has shown how the S315T mutation could block binding of isoniazid without interfering with catalysis [48]. Only one other mutation, the S275T, has been evaluated in mice and has been found to be of low virulence [23]. However, because all of the KatG mutations identified in this study have not been functionally characterized, and some do not abolish enzymatic activity completely, it therefore remains possible that other characteristics linked to both the KatG S315T and *inhA* promoter mutations could account for the differences in transmission between mutants. Nevertheless, our results suggest that only the KatG S315T mutant retains sufficient levels of catalase-peroxidase activity to be effectively propagated within the study setting.

Mutations in the *inhA* gene promoter were also associated with secondary cases. There is no obvious explanation of how *inhA* promoter mutations might affect pathogenicity, but they have not been formally evaluated in animal models. Mutations intrinsic to the *inhA* gene have been formally tested and do not affect pathogenicity [49]. Mutations in the promoter of *ahpC* are thought to compensate for the loss of KatG activity in isoniazid-resistant strains through up-regulation of *ahpC* that encodes a putative alkyl hydroperoxide reductase [32–34]. This notion is supported by our study, as ten out of 11 isolates with *ahpC* promoter mutations were found to have mutations or deletions in *katG.* None of these double mutants were genetically clustered, indicating that *ahpC* promoter mutations are not essential or important for the transmission of isoniazid-resistant strains. Other genetic mechanisms of resistance to isoniazid exist that were not examined here, but these occur infrequently in clinical isolates and are therefore unlikely to be the basis for our results [26].

Mutational events are generally random and individual tuberculosis patients can harbor subpopulations of the same mycobacterial clone with different drug resistance–conferring mutations [50]. However, studies have shown that there seems to be a strong selection for low-cost drug resistance mutations in vivo [10,12,51]. The fact that the KatG S315T and the *inhA*-15;c-t mutations were associated with secondary cases in San Francisco suggests that selection for low-cost mutations in humans may also occur through successful transmission. This is consistent with other studies reporting these two mutations as the clinically most common isoniazid resistance mutations [27,52,53].

An obvious question posed by these results is how the KatG S315T and *inhA* promoter mutants compare to fully susceptible strains. A number of potential confounders make comparisons between resistant and drug-sensitive strains problematic [51,54]. For example, the use of additional genetic and phenotypic markers to epidemiologically link the drug-resistant strains will reduce cluster size in this group. Nevertheless, despite the effective tuberculosis control program in San Francisco [21], the KatG S315T and *inhA* promoter mutants were still transmitting and causing secondary cases of drug-resistant M. tuberculosis. Therefore, in less successful programs, or in areas where resistance testing and individualized treatment are not available, a longer period of infectiousness of patients will result in dissemination of these strains.

In addition to showing that drug resistance mutations are affecting pathogenicity, our results demonstrate a second means by which bacterial genetics are influencing the emergence of drug-resistant tuberculosis. We found that different strain lineages were associated with distinct isoniazid resistance mutations. These lineages are dominant in specific geographical areas where the patients are likely to have acquired their tuberculosis infection [45,46]. Differing tuberculosis control and patient adherence practices in each region could favor the emergence of specific drug resistance mutations. However, this seems unlikely as the East-Asian strains were from patients originally from a geographically wide area, including Vietnam, China, and Hong Kong, throughout which treatment practices will have varied. An alternative explanation is that there are genetic differences between the strain lineages that influence their adaptation to isoniazid, as has been observed in other bacteria [11]. For example, the association of the East-Asian lineage, with KatG mutations predicted to be poorly functional, suggests this lineage may be better able to adapt to increases in oxidative stress. Interestingly, a recent study has shown that members of the East-Asian strain lineage produce a phenolic glycolipid that inhibits the innate immune response [55], which might affect the oxidative burst generated by host immunity [28].

Other studies have failed to demonstrate similar associations between specific drug resistance mutations and strain genotypes [56]. This discrepancy is most likely due to the different genetic markers used to define the different mycobacterial lineages. Traditional mycobacterial genotyping techniques used in epidemiological studies of tuberculosis transmission are based on mobile or repetitive genetic elements that change relatively rapidly [57], accounting for the high discriminatory power of these techniques. However, because of this fast molecular clock, similar DNA patterns can emerge in phylogenetically unrelated strains (homoplasy), making it difficult to infer deep phylogenetic groups unambiguously [46,58–61]. To our knowledge, only one other study has used unique event polymorphisms to define strain lineages in a large collection of drug-resistant strains. In their study, Baker and colleagues defined major strain lineages using synonymous single nucleotide polymorphisms [47]. Consistent with our findings, Baker and co-workers reported the same association between the Indo-Oceanic lineage (corresponding to Lineage IV in the study by Baker et al.) and the *inhA* promoter-15c-t mutation. No association with the East-Asian lineage was found in the study by Baker et al., probably because of the limited number of East-Asian strains that were included [47].

The study presented here is based on the assumption that genetic clustering is an accurate measure of ongoing tuberculosis transmission. Although there is a wealth of published evidence in support of this [25,36–41], alternative hypotheses can be envisaged. For example, certain combinations of strain genetic background and resistance mutations could result in hyper-virulence leading to rapid progression and a greater probability of diagnosis and inclusion in the study. This cannot be ruled out, but is unlikely, as the transmission dynamics of the three main genetic lineages in San Francisco have been studied and considered here [45]. Furthermore, 85% of all isoniazid-resistant isolates collected during a 9-y period by population-based sampling were included in this study, so systematic exclusion of specific genotypes is also unlikely.

In conclusion, our study shows that in addition to well-described clinical determinants of tuberculosis transmission, bacterial genetics also have an important impact on the propagation of drug-resistant M. tuberculosis. Further work is required to establish whether these findings can be extended to other classes of anti-tuberculosis drugs and to other settings such as those with high rates of transmission where host susceptibility is altered by HIV infection. Ultimately, a complete understanding of drug resistance will require the integration of host factors, patterns of antibacterial usage, as well as bacterial genetics.

## Materials and Methods

### Study population.

The epidemiological methods and study population have been described previously [21,25]. All patients from San Francisco who met the Centers for Disease Control and Prevention criteria as an incident case of tuberculosis during the years 1991–1999 were included in the study population. There were 2,498 tuberculosis cases reported, 2,142 (85.7%) were culture positive, and 2,104 (98.2%) were identified as M. tuberculosis.

### Drug resistance testing.

All M. tuberculosis isolates from incident cases of tuberculosis were tested for drug susceptibility using standard BACTEC methods. The following concentrations were used to define resistance: isoniazid 0.1 mg/l, rifampicin 2 mg/l, streptomycin 2 mg/l, and ethambutol 2.5 mg/l. When detected, drug resistance was confirmed with culture on solid media. Patients infected with strains that were initially fully susceptible, but who subsequently developed resistant strains, were not included in this study.

### Molecular epidemiology.

Standard epidemiologic data were collected as part of a prospective population-based molecular epidemiologic study of tuberculosis in San Francisco [25]. For each isolate, genomic DNA was extracted from 10-ml liquid cultures and sub-cultured from the initial Lowenstein Jensen slope used for the primary isolation. RFLP typing was performed following standardized procedures [57], with patterns entered and analyzed using a BioImage Whole Band Analyzer software (version 9 3.3, BioImage Corporation, Ann Arbor, Michigan, United States). Isolates with fewer than six copies of IS*6110* were further genotyped using polymorphic GC-rich sequence (PGRS) RFLP [57]. For the majority of isolates an aliquot of this original DNA was preserved at −20 °C and was used as a template for amplifying resistance genes. In a few cases isolates were re-cultured from −80 °C stocks preserved in glycerol. The protocols and the procedures for the protection of human participants were approved by Stanford University and the University of California, San Francisco.

Isolates with the same RFLP genotype (± one IS*6110* band for strains with > 5 bands; ± one IS*6110* band and identical PGRS patterns in isolates with < 6 IS*6110* copies), the same drug resistance profile, the same drug resistance–conferring mutation, and belonging to the same M. tuberculosis lineage were considered genetically clustered and part of a chain of transmission. Isolates that differed in any of these criteria were considered unique and not linked epidemiologically. Two isolates were classified as clustered based on the RFLP pattern and drug resistance profile.

### Sequencing of drug resistance genes.

To determine the mutations responsible for isoniazid resistance in each isolate, known resistance genes were sequenced. Oligonucleotide primers were designed for the amplification and sequencing of the entire *furA*-*katG* locus (H37Rv: 2153626–2156657, 3,031 bp), the promoter region of *inhA* (H37Rv: 1673196–1673653, 457 bp), and the *oxyR*-*ahpC* intergenic region (H37Rv: 2726390–2725888, 502 bp). DNA was amplified in a 25-μl reaction using a GeneAmp system 9700 thermocycler (Applied Biosystems, Foster City, California, United States). Unincorporated nucleotides and primers were removed by filtration with Multiscreen-PCR plates (Millipore, Billerica, Massachusetts, United States). Sequencing reactions were performed with the ET Terminator sequencing kit (Amersham Biosciences, Little Chalfont, United Kingdom) and purified in Multiscreen plates (Millipore) with Sephadex (Amersham Biosciences). Sequence data were generated with an ABI 377 automated sequencer (Applied Biosystems) at the PAN facility, Stanford University (http://cmgm.stanford.edu/pan) and analyzed with SeqMan (DNASTAR, Inc.).

### Determination of strain lineage by multiplex real-time PCR.

The population structure of M. tuberculosis in San Francisco consists of three main phylogenetic lineages [45]. To determine if the study isolates belonged to either the East-Asian lineage, the Indo-Oceanic lineage, or the Euro-American lineage, the corresponding lineage specific markers were targeted by multiplex real-time PCR as reported previously [45,62,63]. The three lineages were defined based on the absence of the region of difference (RD) 105, absence of RD239, and the ctg to cgg substitution at KatG463, respectively.

### Statistical analysis.

To explore the relationship between RFLP clustering and clinical, demographic, and bacterial variables, univariate analyses using the χ^2^ test of proportions or the 2-tailed Fisher's exact test and multivariate logistic regression modeling were performed. All variables with a *p*-value ≤ 0.2 in the univariate analysis and biological plausibility were considered for the multivariate logistic regression model. We performed forward stepwise model construction and compared the log likelihood ratios of successive models until the final, most parsimonious model was identified [64]. We also tested the significance of potential interaction terms. Statistical analyses were performed with Stata (version 7E; Stata Corporation, College Station, Texas, United States).

## Abbreviation

RFLP: restriction fragment length polymorphism
